# Transmission of *Staphylococcus aureus* between health-care workers, the environment, and patients in an intensive care unit: a longitudinal cohort study based on whole-genome sequencing

**DOI:** 10.1016/S1473-3099(16)30413-3

**Published:** 2017-02

**Authors:** James R Price, Kevin Cole, Andrew Bexley, Vasiliki Kostiou, David W Eyre, Tanya Golubchik, Daniel J Wilson, Derrick W Crook, A Sarah Walker, Timothy E A Peto, Martin J Llewelyn, John Paul, Modernising Medical Microbiology informatics group

**Affiliations:** a Department of Microbiology and Infection, Royal Sussex County Hospital, Brighton, UK; b Public Health England, Royal Sussex County Hospital, Brighton, UK; c Nuffield Department of Clinical Medicine, Experimental Medicine Division, John Radcliffe Hospital, Oxford, UK; d National Institute for Health Research Oxford Biomedical Research Centre, John Radcliffe Hospital, Oxford, UK; e Public Health England, London, UK; f Division of Medicine, Brighton and Sussex Medical School, Falmer, UK

## Abstract

**Background:**

Health-care workers have been implicated in nosocomial outbreaks of *Staphylococcus aureus*, but the dearth of evidence from non-outbreak situations means that routine health-care worker screening and *S aureus* eradication are controversial. We aimed to determine how often *S aureus* is transmitted from health-care workers or the environment to patients in an intensive care unit (ICU) and a high-dependency unit (HDU) where standard infection control measures were in place.

**Methods:**

In this longitudinal cohort study, we systematically sampled health-care workers, the environment, and patients over 14 months at the ICU and HDU of the Royal Sussex County Hospital, Brighton, England. Nasal swabs were taken from health-care workers every 4 weeks, bed spaces were sampled monthly, and screening swabs were obtained from patients at admission to the ICU or HDU, weekly thereafter, and at discharge. Isolates were cultured and their whole genome sequenced, and we used the threshold of 40 single-nucleotide variants (SNVs) or fewer to define subtypes and infer recent transmission.

**Findings:**

Between Oct 31, 2011, and Dec 23, 2012, we sampled 198 health-care workers, 40 environmental locations, and 1854 patients; 1819 isolates were sequenced. Median nasal carriage rate of *S aureus* in health-care workers at 4-weekly timepoints was 36·9% (IQR 35·7–37·3), and 115 (58%) health-care workers had *S aureus* detected at least once during the study. *S aureus* was identified in 8–50% of environmental samples. 605 genetically distinct subtypes were identified (median SNV difference 273, IQR 162–399) at a rate of 38 (IQR 34–42) per 4-weekly cycle. Only 25 instances of transmission to patients (seven from health-care workers, two from the environment, and 16 from other patients) were detected.

**Interpretation:**

In the presence of standard infection control measures, health-care workers were infrequently sources of transmission to patients. *S aureus* epidemiology in the ICU and HDU is characterised by continuous ingress of distinct subtypes rather than transmission of genetically related strains.

**Funding:**

UK Medical Research Council, Wellcome Trust, Biotechnology and Biological Sciences Research Council, UK National Institute for Health Research, and Public Health England.

## Introduction

*Staphylococcus aureus* is a common commensal bacterium but also a leading cause of health-care-associated infection. Colonisation usually precedes infection, and the risk of invasive disease is greatest immediately after acquisition of a new strain.^1^, ^2^ *S aureus* is transmissible between patients, particularly in high-dependency settings.^3^, ^4^, ^5^, ^6^ Hospitals invest considerable efforts in prevention of direct patient-to-patient transmission or transmission via staff and the environment.^7^ Even with good infection control practice, transmission still occurs.^8^ Colonised health-care workers have been implicated as sources of transmission in outbreaks,^9^, ^10^, ^11^ but the use of health-care worker screening and *S aureus* eradication as routine control measures is controversial.

In this study, we aimed to determine how often *S aureus* is transmitted from health-care workers or the environment to patients in a non-outbreak situation, using whole-genome sequencing to establish genetic relatedness and infer recent transmission.

## Methods

### Study design and participants

In this longitudinal cohort study, we sampled health-care workers, the environment, and patients at the adult intensive care unit (ICU) and high-dependency unit (HDU) of the Royal Sussex County Hospital, a large acute hospital in Brighton, England. Since the rate of transmission between groups was unknown, we conducted the study for 14 months (from Oct 31, 2011, to Dec 23, 2012) and did not prespecify fixed sample sizes. The ICU has one five-bedded area, one four-bedded area, three double side-rooms, and one single side-room. The HDU, two floors below the ICU, has two four-bedded areas, one two-bedded area (opened in April, 2012), and two single side-rooms. During the study, infection control practice followed UK National Health Service Guidelines (panel).^12^

**Research in context**

**Evidence before this study**

We searched PubMed from April 1, 2001, to Sept 1, 2016, for studies published in any language using the terms “*Staphylococcus aureus*”, “MRSA”, “healthcare worker or staff”, “transmission”, “whole-genome sequencing”, and “molecular epidemiology”. We found two published reports documenting the use of whole-genome sequencing and describing the involvement of health-care workers in *S aureus* outbreaks. The first report implicated a health-care worker in a persistent meticillin-resistant *S aureus* (MRSA) outbreak in a neonatal intensive care unit (ICU). In the second study, the investigators characterised two outbreaks of meticillin-susceptible *S aureus* (MSSA) in separate neonatal ICUs and identified a health-care worker who was colonised with highly related strains during each outbreak. Most published reports of *S aureus* transmission in health-care settings focus on MRSA and the investigation of outbreaks. To our knowledge, no prospective, whole-genome sequencing-based studies of nosocomial carriage and transmission of *S aureus* in the endemic setting have been done.

**Added value of this study**

In this study, we systematically sampled health-care workers, the environment, and patients in a critical care unit over 14 months and used whole-genome sequencing to characterise all available isolates of *S aureus*, thus providing a comprehensive description of colonisation and transmission. Quite unexpectedly, we found continuous ingress of genetically diverse *S aureus* strains with little onward transmission, despite high rates of carriage by health-care workers and patients, and in the environment.

**Implications of all the available evidence**

In settings where good infection control practice is in place, additional measures to prevent *S aureus* transmission are unlikely to reduce infection rates. Many acquisitions might be attributable to recrudescence of cryptic carriage and should not be routinely ascribed to transmission and breached infection control. We present methods that are of value to those involved in the development and use of methods based on whole-genome sequencing to investigate *S aureus* colonisation and transmission. The possibility that patients experience recrudescence of cryptic *S aureus* carriage requires further investigation to understand its mechanism and develop strategies to identify and protect patients from invasive disease.

In this study, patient sampling and data collection without individual consent were approved by the Research Ethics Committee (10/H0505/83) and Health Research Authority (ECC 8-05 (e)/2010), and screening and data collection of health-care workers were approved by the Research Ethics Committee (11/LO/1451). All health-care workers provided written informed consent.

### Procedures

We invited all nurses, doctors, and physiotherapists with direct patient contact in the ICU or HDU, including temporary staff, to participate in the study. Following successful recruitment of nurses from Oct 31, 2011, doctors and physiotherapists were additionally recruited from April 16, 2012. Nasal swabs were taken from participating health-care workers every 4 weeks (give or take 1 week). Demographic characteristics, comorbidities, and risk factors for *S aureus* carriage were collected through anonymised questionnaires completed with each sample (see appendix for anonymisation details). Separate consent was sought for three substudies investigating transient carriage (nasal swabbing before and after shifts during 2 working days), multiple anatomical site carriage (additional swabs from the throat, axillae, groin, and any broken skin), and throat carriage (additional throat swabs every 6 months).

We sampled each bed space monthly by swabbing three sites of frequent staff contact (monitor button, wipe-clean keyboard, and disposable curtain) and two less accessible sites (floor behind bed and underside of bed). The blood gas machine in a central utility room was swabbed monthly. Air samples were taken from ten sites monthly (plus an additional site from month 6 for two newly opened HDU bed spaces) with an intake of 100 L/min and an impact speed of less than 20 m/s (airIDEAL, Biomerieux, Marcy-l'Étoile, France), as recommended by the International Organization for Standardization.^13^ Monthly environmental sampling by the research team followed clinical rounds (mid-morning) to minimise effect on clinical duties. Patients were present in bed spaces during sampling. Disposable curtains were changed after patient discharge but not immediately before sampling. Clinical areas were cleaned continuously, but study sites were not cleaned immediately before sampling.

As per routine clinical practice, nasal swabs were obtained from all patients at admission to the ICU or HDU (usually within 24 h), weekly thereafter, and at discharge. Perineal swabs were also taken for most patients. All available screening swabs were included in the study and cultured for meticillin-resistant *S aureus* (MRSA) and meticillin-susceptible *S aureus* (MSSA). All patients admitted to the ICU or HDU were eligible and included if they were screened. Additionally, to optimise detection of transmission to patients, from March 1, 2012, sputum, respiratory, urine, wound, and blood culture samples taken for diagnostic purposes yielding *S aureus* were included. Anonymised data regarding the patient, hospital stay, and ICU or HDU bed stay were collected from patient records and ICU or HDU routine screen results from the laboratory database.

All isolates were cultured with SaSelect (Oxoid, Basingstoke, UK) and MRSAselect chromogenic agar plates (Bio-Rad, Redmond, WA, USA). Since the nosocomial population structure of *S aureus* comprises a small number of lineages within which closely related strains cannot be reliably distinguished by conventional molecular typing techniques,^8^ we used whole-genome sequencing (Illumina HiSeq2500, Illumina Inc, San Diego, CA, USA) to establish genetic relatedness of study isolates (appendix). To enhance detection of *S aureus*, swabs from health-care workers and environmental samples underwent broth enrichment.

### Statistical analysis

All analyses were done at the level of *S aureus* subtype, which was used to infer compatibility with involvement in recent direct or indirect transmission.^8^, ^14^ Isolates were defined to be of the same subtype if they differed by no more than 40 single-nucleotide variants (SNVs), equating to roughly 5 years of evolution. To assess diversity of isolates, we calculated the median and maximum SNV differences seen within individual health-care workers across different combinations of sampling sites and time, and the minimum SNV differences seen between health-care workers across the whole study. *S aureus* acquisition was defined by a culture-negative screen followed by a culture-positive screen or diagnostic sample, or by a culture-positive sample followed by a culture-positive sample of a different subtype. Transmission from health-care worker to patient was defined as patient acquisition of a subtype cultured from a health-care worker either at the same time or at any previous timepoint in the study. Patient-to-patient transmission was defined as patient acquisition of a subtype cultured from a previous patient, irrespective of whether they shared time in the ICU or HDU. Transient health-care worker carriage was defined as culture-negative pre-shift nasal screen preceding culture-positive post-shift screen on day one, followed by culture-negative screen on day two.^15^ We did not account for missing samples or failure in whole-genome sequencing. We used medians, IQR, and rank-sum tests to compare continuous data and exact tests to compare categorical data.

### Role of the funding source

The funder of the study had no role in study design, data collection, data analysis, data interpretation, or writing of the report. The corresponding author had full access to all the data in the study and had final responsibility for the decision to submit for publication.

## Results

Of 208 eligible health-care workers, 198 (95%) consented to participate in the study. 73 (37%) were *S aureus* nasal carriers at enrolment, of whom eight (4%) carried MRSA (table). During the study, 115 (58%) health-care workers yielded *S aureus* at least once from nasal swabs. Nasal carriage rates at any timepoint were similar among nurses, doctors, and physiotherapists (p=0·50) and at 4-weekly sampling intervals (median 36·9%, IQR 35·7–37·3; appendix p 12).

**Figure 1:**
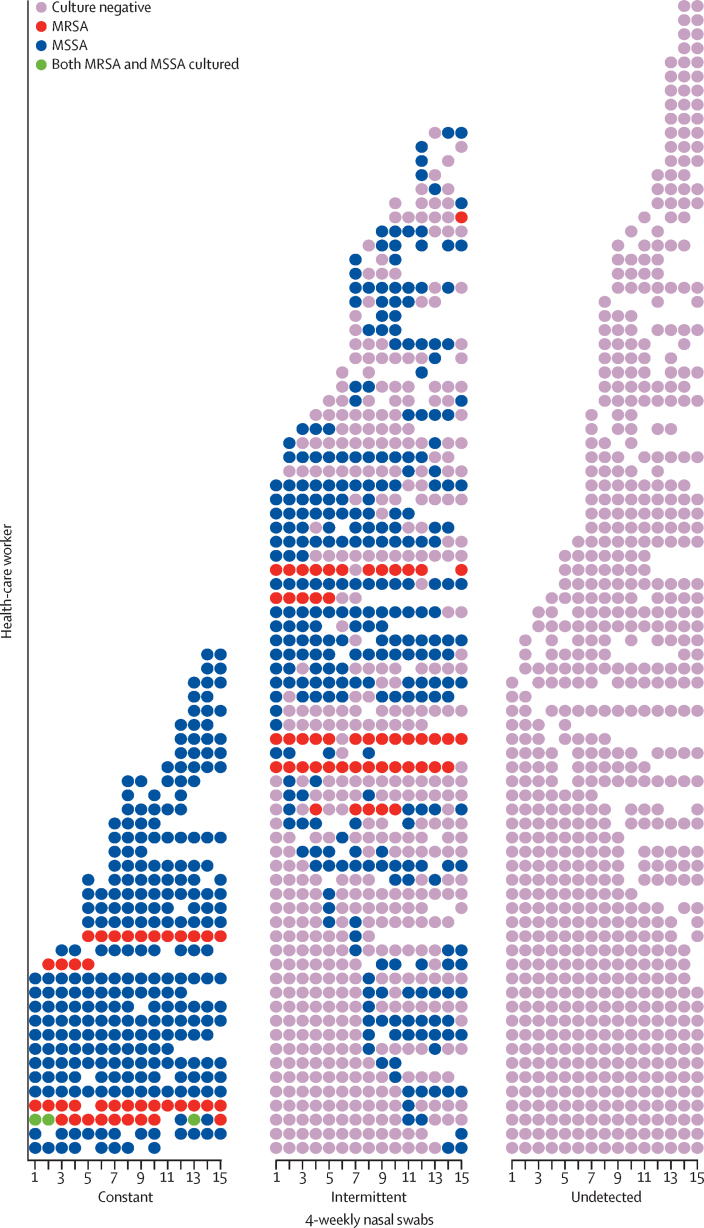
Nasal colonisation of *Staphylococcus aureus* in 191 serially screened health-care workers Each dot represents culture results of 4-weekly nasal swabs. MRSA=meticillin-resistant *S aureus*. MSSA=meticillin-susceptible *S aureus*.

Longitudinal data were available for 191 health-care workers who returned a median of ten (range 2–15) nasal swabs taken every 4 weeks. Three patterns of nasal carriage were observed (figure 1). 82 (43%) health-care workers were always culture negative, 36 (19%) were always culture positive (31 with a single subtype), and 73 (38%) were intermittently culture positive (59 with a single subtype).

In the substudy to assess the sensitivity of nasal swabbing for detection of carriage at any anatomical site, 45 (37%) of 122 nurses were culture positive by nasal swab, 61 (50%) were culture positive when throat swabs were included, and 64 (52%) were culture positive when all body sites were included. Hence, the sensitivity of nasal swabbing was 70·3% (95% CI 57·6–81·1).

To assess whether nursing staff acquire *S aureus* carriage transiently following patient contact, 103 nurses had nasal swabs before and after a shift on day one and before a shift on day two. Concordance of culture results between swabs was 98%. Transient nasal acquisition was not detected.

**Figure 2:**
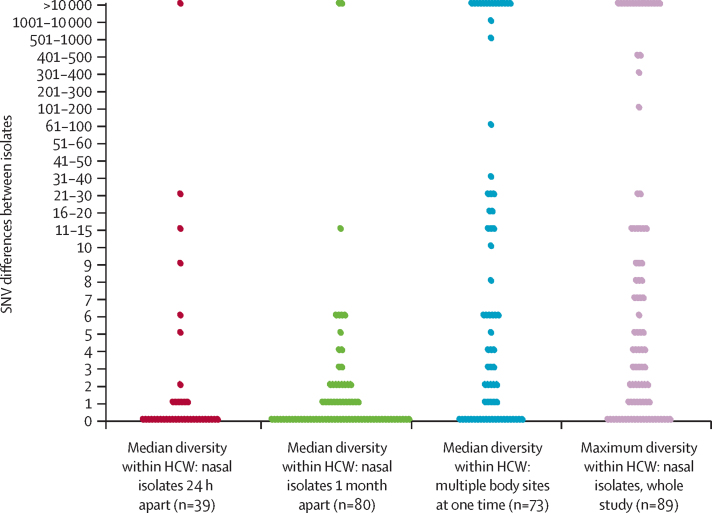
Within-host diversity of *Staphylococcus aureus* in health-care workers Median SNV differences between pairs of nasal isolates cultured 24 h apart and 1 month apart, and between multiple body sites at the same timepoint. Maximum SNV differences in nasal isolates during the entire study. Each dot represents the SNV between two isolates cultured from each health-care worker, and the median is shown when more than one pair was available for each health-care worker. HCW=health-care workers. SNVs=single-nucleotide variants.

**Figure 3:**
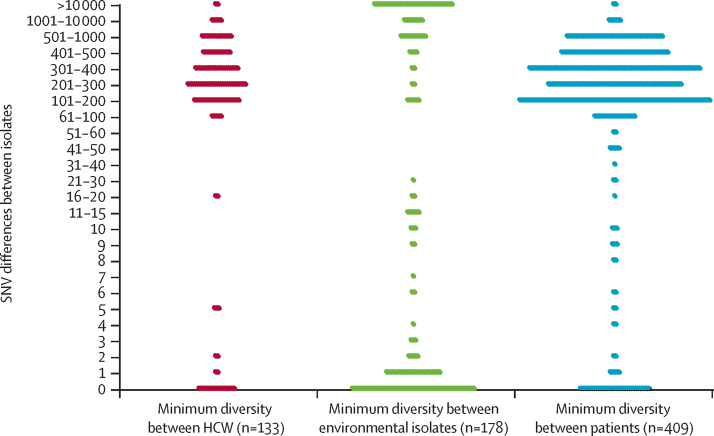
Genomic diversity of *Staphylococcus aureus* isolates obtained from health-care workers, environmental samples, and patients Minimum SNVs between isolates taken from different health-care workers, between environmental isolates and any other environmental isolate cultured in the same month, and between different patients. Each dot represents the SNV difference between two isolates. HCW=health-care workers. SNVs=single-nucleotide variants.

In total, 937 *S aureus* isolates were obtained from health-care workers during the study, and whole-genome sequencing was successfully done for 902 (96%) of these isolates (appendix p 6). 242 (86%) of 281 representative median within-host SNV differences were fewer than 40, indicating the presence of a single subtype across different anatomical sites and over time (figure 2; appendix p 7). The median of the remaining within-host SNV differences (>40) was more than 10 000 and all were at least 75 SNVs apart, indicating subtypes separated by more than 9 years of evolutionary time and consistent with infection by more than one subtype across anatomical sites or over time. By contrast with low within-host diversity, the strains of *S aureus* carried by different health-care workers were predominantly distinct from each other (figure 3). 26 (20%) of 133 isolates differed from another health-care worker isolate by fewer than 40 SNVs, and most of these were within five SNVs, suggesting recent transmission between health-care workers or acquisition in both health-care workers from a common source. Over the 14 month study period, we detected 69 *S aureus* acquisitions involving 54 (28%) of 191 health-care workers. Acquisition was defined by change of colonisation status from culture negative to culture positive for 40 acquisitions and change of subtype for 29 acquisitions (table).

2153 environmental samples (1981 from bed spaces, 15 from the blood gas machine, and 157 from the air) were taken from 40 locations (28 bed spaces, one blood gas machine, and 11 air sampling sites) during the study. *S aureus* was identified in the environment at every 4-weekly sampling timepoint (appendix p 12), with 8% (two of 26) to 39% (11 of 28) of bed space samples and 9–50% (1–5 of ten) of air samples yielding a total of 178 *S aureus* isolates (23 of which were MRSA). The genetic diversity of environmental isolates across the study was bimodal. 107 (60%) of 178 isolates were within 40 SNVs of another environmental isolate (of which 81 [76%] of 107 were within one SNV), whereas 71 (40%) of 178 isolates were more than 100 SNVs (predominantly >1000 SNVs) apart (figure 3).

During the study, 1933 admissions (involving 1760 patients) to the ICU and HDU were recorded, including 20 patients who were already in the ICU or HDU at study initiation, 1889 admitted and discharged, and 24 remaining in the ICU or HDU at study end. Median length of stay was 3·0 days (IQR 1·6–6·0), and median age was 65·5 years (IQR 48·8–76·6). 1164 (60%) admissions involved male patients. 1854 (96%) of 1933 admissions were screened at least once for *S aureus* in the ICU or HDU (appendix p 13), and 1784 (92%) were screened within 24 h of admission. 41 (52%) of 79 unscreened admissions involved patients who had been in the ICU or HDU for 24 h or less. 1127 (61%) admissions were sampled serially (median two [range 2–32] screens per admission).

At admission to the ICU or HDU, 386 (21%) of 1854 screened admissions carried *S aureus*, of whom 39 (2%) carried MRSA (table). 357 (20%) of 1784 patients swabbed within 24 h of admission were culture positive, and 12 (17%) of 70 patients whose first swab was taken within 24–48 h of admission were culture positive; an additional 17 patients yielded *S aureus* from diagnostic samples taken within 48 h of admission but had culture-negative swabs at admission screening. Patients admitted to the ICU or HDU within 24 h of hospital admission were more likely to yield *S aureus* from ICU and HDU admission screens (taken ≤24 h) than were patients admitted to ICU or HDU more than 24 h after hospital admission (269 [24%] of 1131 *vs* 88 [13%] of 653, p<0·0001). Most strains of *S aureus* carried by different patients were distinct (figure 3). However, 56 (14%) of 409 patient isolates were fewer than 40 SNVs apart from another patient isolate, suggesting possible involvement in recent transmission networks.

Among 1127 patient admissions sampled on more than one occasion, 92 (8%) acquired *S aureus* while in the ICU or HDU. A total of 97 *S aureus* acquisitions were detected, of which 68 were from culture negative to culture positive (in 67 patients) and 29 were new subtype acquisitions (in 26 patients, including one who newly acquired; table). 20 acquisitions were identified from diagnostic samples, of which three were from bloodstream infections.

**Figure 4:**
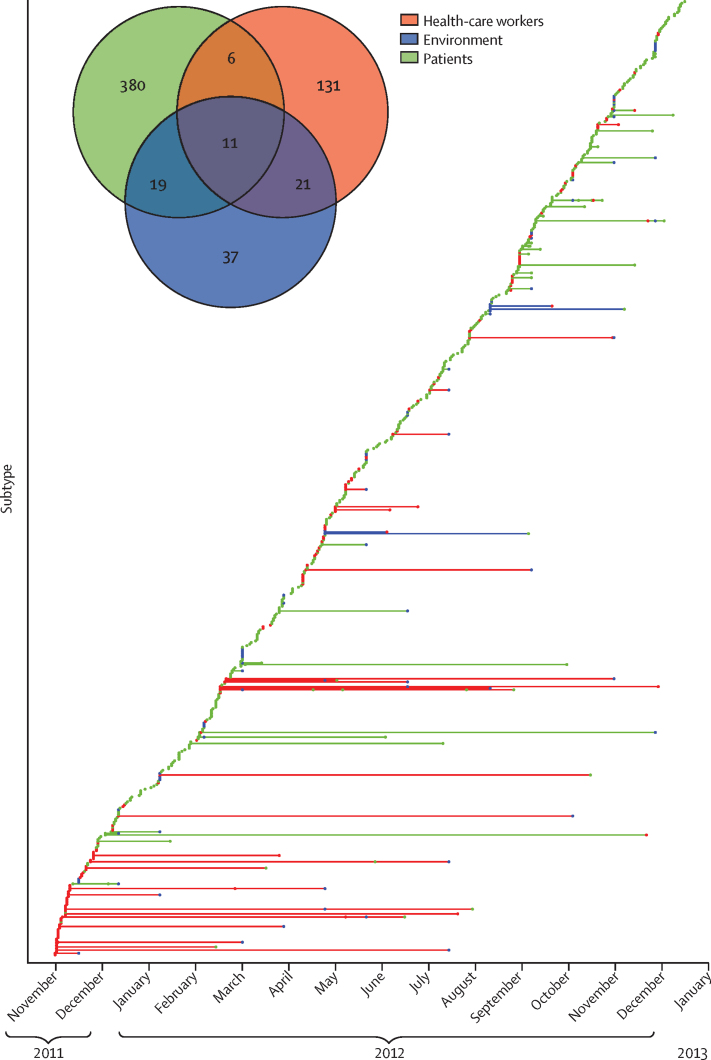
Introduction and source of new *Staphylococcus aureus* subtypes over time The rarefaction curve shows newly identified subtypes (n=605) over time. The first isolate from each subtype and source is plotted according to the date of collection. Where a subtype was retrieved from more than one of the same source (ie, more than one patient or health-care worker), multiple dots of the same colour are plotted. Horizontal lines joining dots of the same subtype are coloured according to the source where each subtype was first identified. The Venn diagram shows sources of individual subtypes identified during the study.

In total, 605 subtypes of *S aureus* were identified during the study. A median of 38 (IQR 34–42) new subtypes was detected per 4-weekly cycle (excluding the first 4 weeks when most health-care workers were recruited; figure 4). The median SNV difference between subtypes across the whole study was 273 (IQR 162–399, range 42–18 171), corresponding to roughly 34–35 years of evolution. The distribution of subtype sizes is shown on appendix p 8. 12 isolates identified using conventional methods as *S aureus* were identified with whole-genome sequencing as *Staphylococcus argenteus* (appendix).

Although 17 (10%) of 169 subtypes identified in health-care workers were also identified in patients, only seven fulfilled criteria for transmission from health-care worker to patient—ie, the newly acquired patient subtype was identified in a health-care worker before or at the same time as the patient (figure 4; appendix pp 9–10). These seven transmissions involved six health-care workers. Five were transmissions of MSSA, and two were of MRSA from the same health-care worker. In the remaining ten matches, four patient isolates at ICU or HDU admission had the same subtype previously found in a health-care worker, suggesting acquisition from a common source outside the unit, and six isolates were identified in the patients before they were first identified in health-care workers, suggesting transmission from patients to health-care workers.

Although 30 (34%) of 88 environmental subtypes were also found in patients, only two were found in the environment and then acquired by a patient (appendix p 11). One subtype was found in a patient at the time of ICU or HDU admission, and the remainder were found in the environment only after they had been identified in patients, suggesting shedding into the environment.

Of the 416 subtypes found in patients, 27 were identified in more than one patient. For 14 subtypes, there were 16 acquisitions where a subtype present or previously present in one patient was acquired by another patient, in addition to the transmissions from health-care workers and the environment (figure 4; appendix p 10). Eight instances where two patients shared the same subtype at the time of ICU or HDU admission were recorded, including six instances where patients had separate ICU or HDU admissions, suggesting a common source outside the unit. For five subtypes, there were six acquisitions where a donor was not identified. Overall, a donor could be identified in 25 (26%) of 97 patient acquisitions.

## Discussion

We have exhaustively investigated *S aureus* transmission in an ICU and an HDU where standard UK infection control measures were in place. We sampled health-care workers, the environment, and patients and used whole-genome sequencing to determine the genetic relatedness of isolates. Using a threshold of 40 SNVs (equivalent to roughly 5 years of evolution) to define genetic subtypes, we detected 605 genetically distinct subtypes separated by a median of 273 SNVs, equating to roughly 34 years of evolution. Strikingly, although a third of health-care workers carried *S aureus,* only seven instances of transmission from health-care worker to patient were detected. *S aureus* was also widely present in the environment, but we only detected two instances of patient acquisition from the environment. Consistent with results from our previous study,^8^ we also detected some instances of patient-to-patient transmission.

Health-care workers might contribute to nosocomial transmission of *S aureus* as a reservoir or as vectors. Patient acquisition of *S aureus* has been associated with overcrowding, understaffing,^16^, ^17^ and close patient contact.^16^, ^18^ Before the development of whole-genome sequencing, conventional typing and epidemiological approaches had implicated health-care workers in nosocomial transmission of *S aureus*, particularly in MRSA outbreaks.^15^, ^19^, ^20^, ^21^, ^22^, ^23^, ^24^ Two published sequencing-based studies^9^, ^25^ included health-care workers in investigation of neonatal *S aureus* outbreaks. Harris and colleagues^9^ implicated a health-care worker colonised with MRSA in an outbreak that persisted despite environmental cleaning. By contrast, although Roisin and colleagues^25^ identified a health-care worker colonised with an outbreak MSSA strain, sequencing analysis suggested acquisition during the outbreak rather than transmission from health-care worker to patient. Using whole-genome sequencing, we assembled a uniquely comprehensive picture of *S aureus* colonisation and transmission in a well defined high-dependency clinical setting. We showed the presence of highly diverse strains and continuous ingress of new subtypes into the unit rather than ongoing transmission of strains between health-care workers, the environment, and patients.

Our study has limitations. It was done in a single hospital, and our findings might not be generalisable to all locations. However, our study setting is likely to be typical, in terms of risk factors for *S aureus* transmission,^3^ of high-dependency care settings elsewhere. Since the median length of stay was short (3 days), 806 (42%) of 1193 patient admissions were omitted from secondary screening and could not be assessed for acquisition. However, transmission from health-care workers or the environment to this subset of patients would need to be disproportionately large to change our findings. We inferred transmission from health-care worker to patient in two instances where they acquired the same subtype of *S aureus* in the same month. However, since health-care workers were screened every 4 weeks, transmission from patient to health-care worker is an alternative interpretation. Because the sensitivity of nasal screening is imperfect, we might have underestimated colonisation rates in patients and health-care workers. Our 4-weekly sampling interval for health-care workers could have missed some transient carriage, although the carriage patterns we observed were strikingly consistent. We also staggered recruitment of different groups of health-care workers, starting with nurses in view of their high patient contact, followed by doctors and physiotherapists after successful study implementation. Nevertheless, our method represents the reality of clinical practice; even if some transmissions from health-care workers to patients were not detected, such additional carriage would have to account for a substantial and disproportionate number of transmissions to change our fundamental observation—namely, in the context of good infection control, patients are not at high risk of *S aureus* acquisition, despite the substantial burden of *S aureus* detected in health-care workers, the environment, and patients.

The high genetic diversity and low level of transmission we observed contrast strikingly with findings from studies in low-income settings with lower barriers to transmission, in which multiple transmissions of small numbers of strains have been documented.^26^ Our findings therefore underscore the effectiveness and importance of measures implemented to prevent nosocomial transmission.

Despite intensive sampling, only 25 (26%) of 97 patient acquisitions in our study could be linked to putative donors. The lower carriage rates for patients compared with health-care workers suggest that a substantial proportion of suspected acquisitions might not actually be true acquisitions. Low levels of colonisation (eg, following antibiotic exposure) might result in a false-negative admission screen. Some acquisitions might thus represent recrudescence from cryptic (eg, intracellular^27^) foci rather than relative insensitivity of screening by swabbing mucosal surfaces. Additional explanations for unattributed acquisitions include unsampled putative sources such as visitors to the ICU or HDU and the food chain.

To conclude, in the presence of robust infection control measures, the critical care setting is characterised by genetically diverse and continually changing patterns of *S aureus* colonisation of patients, staff members, and the environment, but transmission to patients occurs infrequently. Therefore, use of additional measures to reduce *S aureus* colonisation of health-care workers and the environment might provide little extra protection to patients in a non-outbreak situation.

## Figures and Tables

**Table:** Identification of *Staphylococcus aureus* in health-care workers and patients admitted to the intensive care and high-dependency unit

		**Nurses (n=149)**	**Doctors (n=40)**	**Physiotherapists (n=9)**	**Total health-care workers (n=198)**	**Patient admissions (n=1933)**
Age, years						
	16–29	40 (27%)	9 (23%)	8 (89%)	57 (29%)	154 (8%)
	30–39	71 (48%)	17 (43%)	1 (11%)	89 (45%)	150 (8%)
	40–49	28 (19%)	11 (28%)	0	39 (20%)	208 (11%)
	50–59	9 (6%)	3 (8%)	0	12 (6%)	264 (14%)
	≥60	1 (1%)	0	0	1 (1%)	1157 (60%)
Male sex		25 (17%)	24 (60%)	2 (22%)	51 (26%)	1164 (60%)
Nasal carriage at enrolment		54 (36%)	16 (40%)	3 (33%)	73 (37%)	386* (21%)
	MRSA	8 (5%)	0	0	8 (4%)	39 (2%)
Total acquisitions of *S aureus* during study		60	5	4	69	97
	MRSA	3 (5%)	0	1 (25%)	4 (6%)	19 (20%)
Culture negative to positive acquisitions during study		31	5	4	40	68
	MRSA	0	0	1 (25%)	1 (3%)	14 (21%)
Culture positive to new subtype acquisitions during study		29	0	0	29	29
	MRSA	3 (10%)	0	0	3 (10%)	5 (17%)
Acquisition isolates available for whole-genome sequencing		59 (98%)	5 (100%)	4 (100%)	68 (99%)	86 (89%)

Data are n (%) or n. MRSA=meticillin-resistant *S aureus*.

* Of 1854 patients receiving at least one screen.

## Panel Salient features of infection control and antimicrobial stewardship practice during the study period

**Hand hygiene and nursing practice**

• Hand hygiene with alcohol gel or soap and water, with adherence to WHO Five Moments For Hand Hygiene for all staff and visitors
• Monthly audits of hand hygiene practice reported to infection prevention committee of Hospital Trust
• Compliance with dress code (including bare below elbows and no wrist watches) to facilitate hand hygiene
• Aseptic non-touch technique to reduce contamination of wounds and devices
• Nurse-to-patient ratio maintained at 1:1 for ventilated patients and 1:2 for other patients

**Meticillin-resistant *Staphylococcus aureus* (MRSA) surveillance and isolation**

• MRSA screening (nose plus groin or throat and any wound) for all patients admitted to the hospital
• All patients in critical care had additional MRSA screening on admission to the intensive care unit (ICU) or high-dependency unit (HDU), weekly thereafter, and at discharge
• Patients known to be MRSA positive at ICU or HDU admission were nursed in single-room accommodation or with bed-side precautions (gown and gloves) until single-room accommodation became available (with priority for patients with exfoliating skin conditions, large open wounds, MRSA in sputum, or tracheostomy)
• Patients found to be MRSA positive after ICU or HDU admission were nursed in single-room accommodation or with bed-side precautions (gown and gloves) until single-room accommodation became available
• Health-care workers were not routinely screened

***S aureus* suppression**

• All patients received skin washes with 4% chlorhexidine gluconate aqueous solution
• Patients who were MRSA positive additionally received nasal mupirocin (2%)

**Environmental cleaning and decontamination**

• Routine daily cleaning of environmental surfaces with chlorine-releasing solution
• Removal of dust from horizontal surfaces
• Mattresses and beds cleaned daily or if visibly contaminated with chlorhexidine wipes (2%)
• Terminal cleaning of patient environment
• Disposable curtains changed between patients

**Antimicrobial stewardship**

• A Trust-wide policy was in place that minimised inappropriate or excessive antibiotic therapy and prophylaxis, and limited use of glycopeptides, third-generation cephalosporins, and quinolones
• Selective digestive decontamination was not used
