# Supplementary material for: Transmission of Staphylococcus aureus between health-care workers, the environment, and patients in an intensive care unit: a longitudinal cohort study based on whole-genome sequencing
Source: Lancet Infect Dis. 2017 Feb;17(2):207–14. doi: 10.1016/S1473-3099(16)30413-3 (PMC5266793; doi:10.1016/S1473-3099(16)30413-3)
Supplement: Supplementary appendix [file mmc1.pdf]

THE LANCET Infectious Diseases

Supplementary webappendix

This webappendix formed part of the original submission and has been peer reviewed. We post it as supplied by the authors.

Supplement to: Price JR, Cole K, Bexley A, et al. Transmission of *Staphylococcus aureus* between health-care workers, the environment, and patients in an intensive care unit: a longitudinal cohort study based on whole-genome sequencing. *Lancet Infect Dis* 2016; published online Nov 15. http://dx.doi.org/10.1016/S1473-3099(16)30413-3.

Supplementary material

Table of Contents

Methods 2

Health-care worker anonymisation 2

Culture and identification 2

Whole-genome sequencing 2

Statistical analysis 3

Supplementary data 4

Isolates identified as *Staphylococcus argenteus* 4

Intensive sampling of patients 4

Figure S1. Flow chart depicting *Staphylococcus aureus* isolates and genomes evaluated in the study 6

Figure S2. Maximum within host diversity of *Staphylococcous aureus* in health-care workers 7

Figure S3. Size distribution of Staphylococcus aureus subtypes 8

Figure S4. *Staphylococcus aureus* subtypes first identified in health-care workers 9

Figure S5. *Staphylococcus aureus* subtypes first identified from patients 10

Figure S6. *Staphylococcus aureus* subtypes first identified from an environmental sample 11

Table S1. Monthly prevalence of *Staphylococcus aureus* in health-care workers and environment 12

Table S2. Routine screens from each patient-admission to the intensive care or high dependency unit 13

References 14

Methods

Health-care worker anonymisation

Consenting health-care workers (HCWs) were allocated a unique participant code (UPC) to unlink the subject information and samples from the individual participant. The ward matron (and staff delegates) held the UPC conversion key on a Trust computer with password protection. If any participant misplaced their UPC they could retrieve it from the ward matron (or staff delegate). The conversion key was not known to members of the research team ensuring complete anonymity for staff. Following completion of the study the ward matron destroyed the UPC conversion to ensure non-reversible identification of HCW participant data.

Culture and identification

Patient screening swabs (Amies-Coal plastic stem swab, Sarstedt, Germany) were inoculated onto SaSelect chromogenic agar (Oxoid Ltd, Basingstoke, UK) and chromogenic MRSAselect™ (Bio-Rad, Redmond, USA) agar plates, incubated at 35-37°C in air for 18 hours. To enhance detection of *S. aureus*, HCW and environmental swabs underwent broth enrichment. Swab tips were placed in 7·0% salt broth (Oxoid, Basingstoke, UK) overnight at 35-37°C. A 5µl loop of broth was inoculated onto SaSelect agar. SaSelect agar was used for air sampling. All presumptive *S. aureus* were confirmed with PROLEX™ Staph Xtra Latex Kit (Pro-Lab Diagnostics, Cheshire, UK) and Microflex™ series MALDI-TOF (Bruker Daltonics). Antibiotic susceptibilities were determined by disk diffusion according to BSAC standards.[1] Where two or more distinct colonial morphologies or antimicrobial susceptibility profiles were observed, isolates with each phenotype were subcultured and independently sequenced. Otherwise, a sweep of bacterial colonise was sequenced (Supplementary Figure 1).

Whole-genome sequencing

All available isolates underwent whole-genome sequencing (WGS). Cultures were incubated overnight on individual Columbia Blood Agar (Oxoid, Basingstoke, UK). DNA was purified from a 5µl loop of culture growth using QuickGene DNA tissue kits (Autogen, USA) following manufacturer's instructions. Bacterial libraries were sequenced at the Wellcome Trust Centre for Human Genetics, Oxford, using Illumina HiSeq2500. Paired reads were mapped to the MRSA252 reference genome[2] (CC30) using Stampy 1.0.21-23.[3] Sequences were excluded if <70% of the genome was identified. Single nucleotide variants (SNV) were identified across all mapped non-repetitive sites using SAMtools[4] requiring a consensus of >75% across all reads and minimum read-depth of ≥5 (at least one in each direction). Sequences with >40 variable sites were excluded if (i) the

variable sites were not identified (as either wild-type or mutant) in ≥70% samples (to account for sites variably present/absent which could reflect mobile element or non-reliably called regions) or (ii) <70% of the remaining variable sites were identified (to identify contamination with mixed samples). ClonalFrameML[5] was used to determine pairwise SNV differences from maximum likelihood phylogenies adjusted for recombination. Where WGS data suggested the sample contained >1 genotype, multiple (between 2-6) colonies were re-sequenced (Supplementary Figure 1).

Statistical analysis

Data were analysed using R (version 2·15·3, RStudio, USA) and Stata (version 13·1, StataCorp®, USA). Continuous data were compared using medians, IQR and rank-sum tests, and categorical data using exact tests.

Supplementary data

Isolates identified as Staphylococcus argenteus

Of 1819 isolates successfully sequenced in this study, 12 were genetically highly unrelated to all other study isolates (minimum 191,579 SNV from any other study isolate). The isolates were obtained from four HCWs and two environmental samples. The 12 isolates had been identified using conventional methods (see above) as *S. aureus*. *S. aureus* is not considered to be a highly diverse taxon.[6,7] 16S ribosomal RNA (rRNA) sequences were closely related to a *S. aureus* reference 16S rRNA sequence.[2] To evaluate these isolates further we undertook phylogenetic comparisons with sequenced types of all described taxa in the genus *Staphylococcus*. Whilst 88·5-92·8% of raw reads mapped to a *S. aureus* reference genome, a higher proportion (91·1-97·6%) mapped to the reference genome of *Staphylococcus argenteus*.[8] *S. argenteus* is a recently described clinically significant species, previously assigned to *S. aureus* clonal complex 75.[9-11] *S. argenteus* has identical 16S ribosomal RNA to *S. aureus*[11,12] but in contrast lacks staphyloxanthin (colonies appear silver rather than golden yellow) and possesses a mobile clustered regularly interspaced short palindromic repeat (CRISPR) element inserted into *orfX* gene.[8]

Six *S. argenteus* isolates were highly related (≤15 SNV); 4 (3 nose and 1 throat) isolates from one nurse and two isolates from separate bed spaces cultured in the same month. Three other HCWs yielded *S. argenteus*. A doctor yielded highly-related isolates from serial nasal screens and a throat swab (n=4) and two nurses yielded *S. argenteus* from individual nasal swabs. HCW isolates differed by >100 SNV from isolates cultured from other HCWs. All *S. argenteus* isolates are included in the main results for *S. aureus*.

Intensive sampling of patients

To assess the performance of routine patient screening using nasal and perineal swabs we evaluated two alternative sampling methods in a subset of patients. Firstly, we compared culture results for screens taken routinely by nursing staff with those taken by a dedicated research nurse. The research nurse performed screening on Mondays for 5 consecutive weeks between 1st October and 4th November 2012. Routine ward staff performed screening at other times during the week. Secondly, we compared routine culture methods (direct agar plate inoculation) with adjunctive broth enrichment in patient samples over a 4-week period (3rd-23rd December 2012). All HCW and environmental samples were always cultured following broth enrichment.

There was no evidence of difference in the mean proportion of positive swabs taken by ward nurses (176/1073, 16·4%) versus the research nurse (20/146, 13·7%) (incidence rate ratio (IRR) from Poisson regression

comparing routine staff to research nurse periods = 0·84, 95% CI 0·48-1·46, p=0·54), nor obtained using broth enrichment (24/99, 24·2%) versus direct plating (176/1073, 16·4%) (IRR broth enrichment vs. direct plating = 1·42, 95%, CI 0·82-2·46, p=0·22). Although the differences observed in these substudies were compatible with chance, 95% confidence intervals were relatively wide and therefore we cannot exclude the possibility that both sampling by the research nurse and use of a direct plating method could have led to some under-detection of *S. aureus* in patients.

Figure S1. Flow chart depicting *Staphylococcus aureus* isolates and genomes evaluated in the study

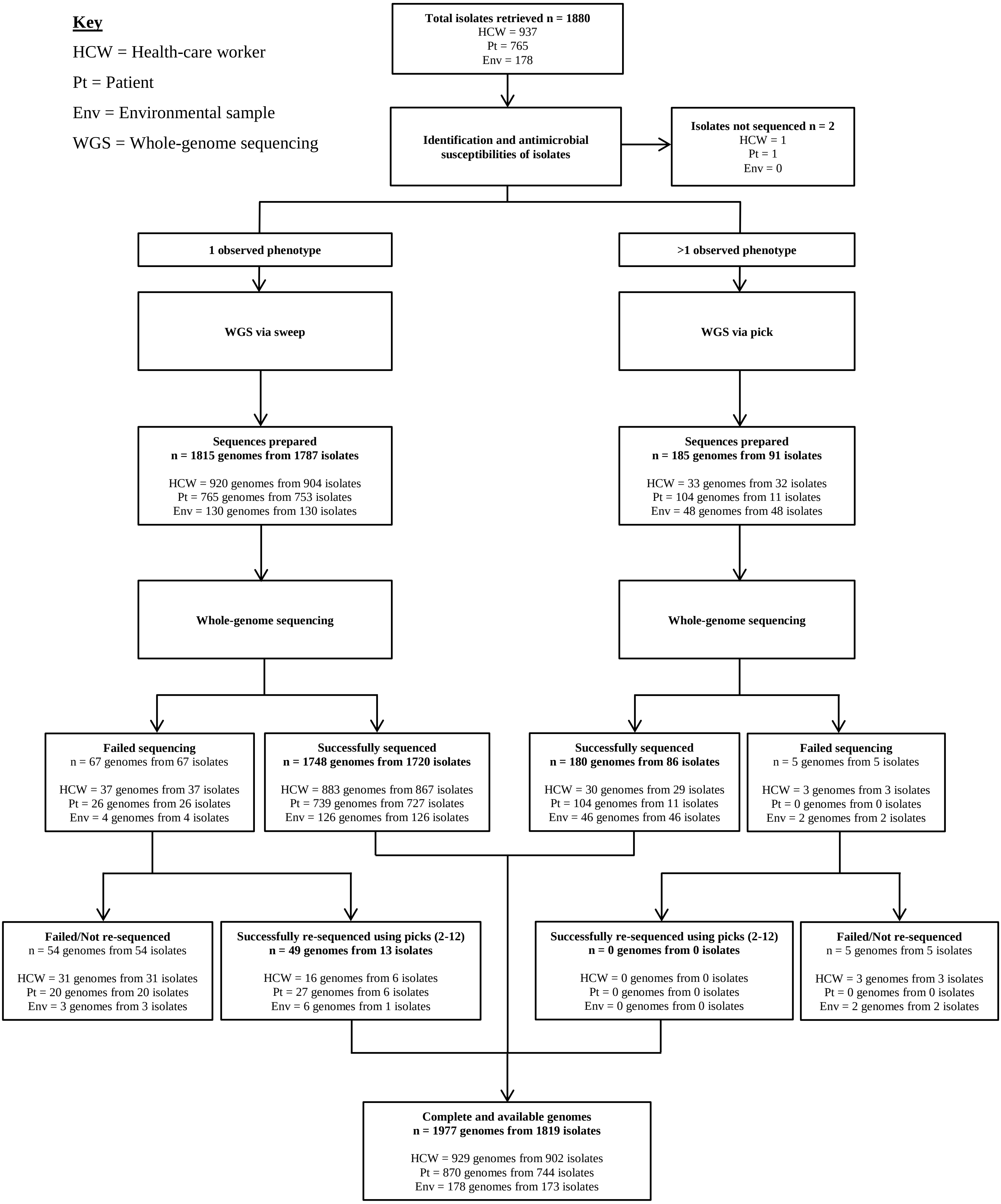

Figure S2. Maximum within host diversity of *Staphylococcous aureus* in health-care workers

The maximum single nucleotide variants (SNV) in a single health-care worker (HCW) between pairs of nasal isolates cultured within 24 hours (A) or one month (B), or between multiple body sites at the same time (C). Each dot represents the SNV between two isolates cultured from a single HCW.

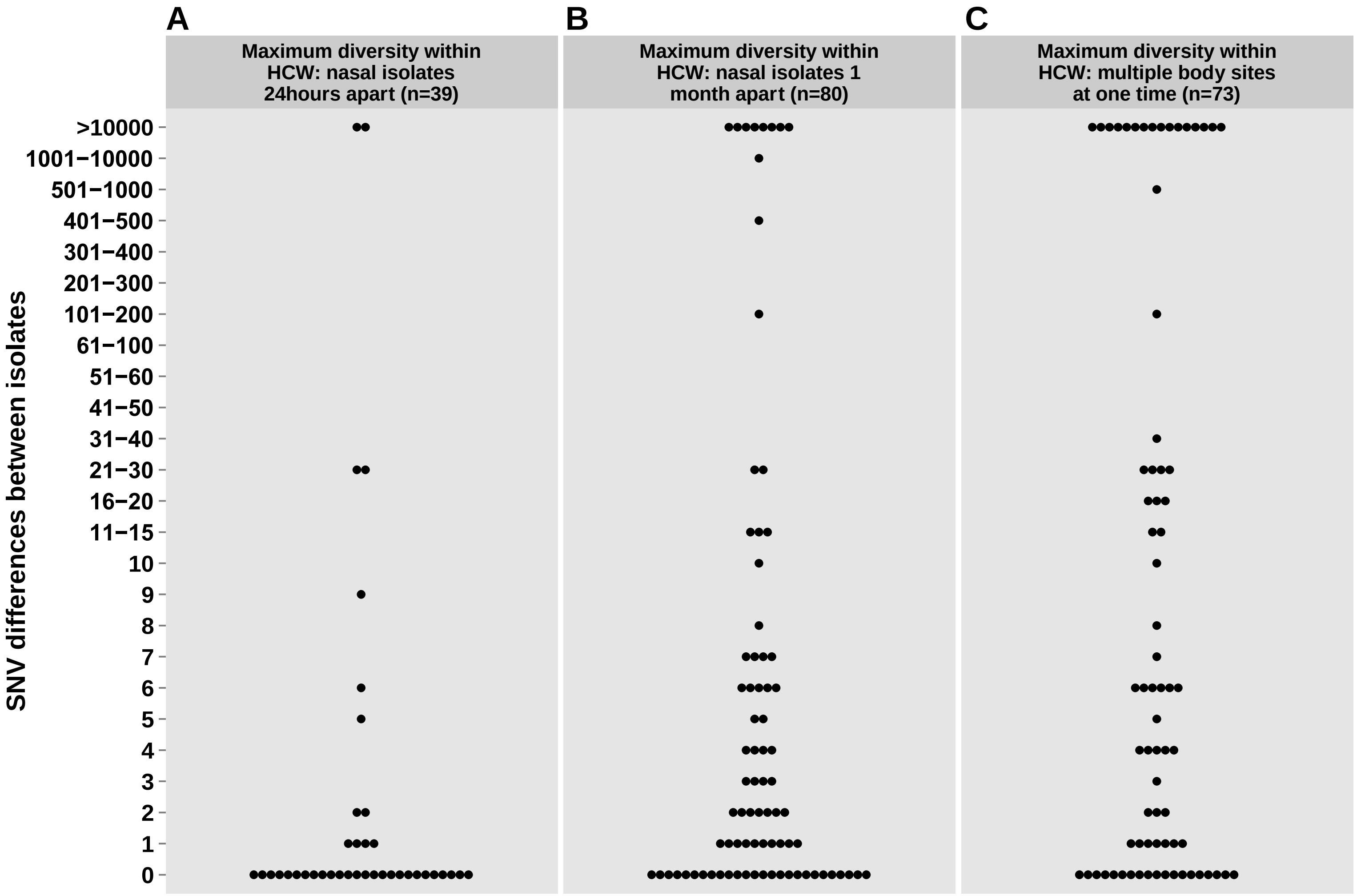

Figure S3. Size distribution of Staphylococcus aureus subtypes

Graph A displays all subtypes (defined as ≤40 single nucleotide variants) containing isolates retrieved from health-care workers (HCWs) or patients (n=568). Subtypes containing environmental isolates are depicted. Graph B displays all subtypes containing isolates retrieved from the environment (n=88). Subtypes containing isolates from human sources (HCWs, patients) are depicted. Subtype size represents the total number of different sources (HCWs, patients, environmental sampling) in which each subtype was identified.

A

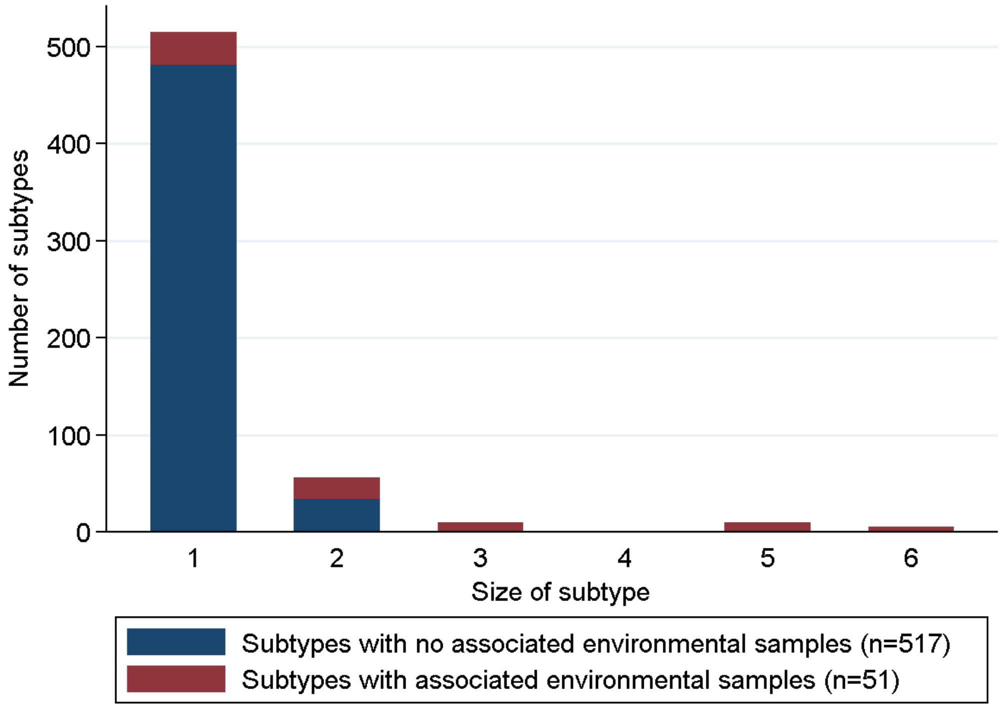

B

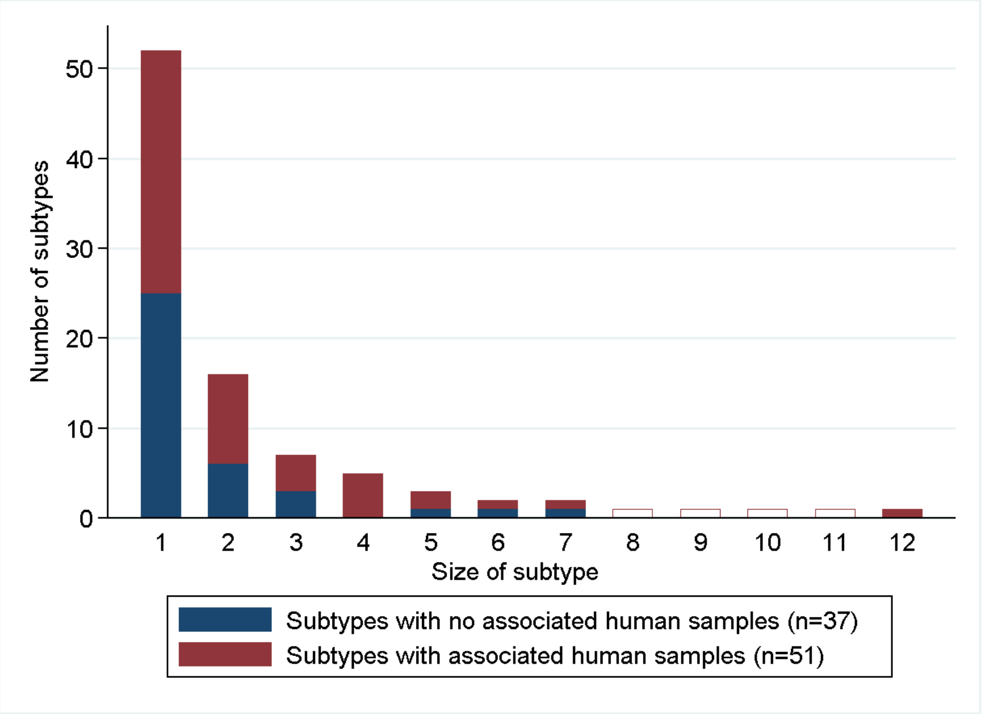

Figure S4. *Staphylococcus aureus* subtypes first identified in health-care workers

Each row represents a subtype first identified in a health-care worker (HCW) (n=157). The first isolate from each subtype and source is plotted according to date of collection. Isolates are coloured according to source; HCW (red), patient (black), and environment (blue). Where a subtype was retrieved from >1 of the same source (i.e. >1 patient or HCW) multiple dots of the same colour are plotted. The horizontal lines joining dots of the same subtype are coloured according to the source where each subtype was first identified; HCW = red, environment = blue, patient = black. Subtypes involving patients (n=9) are labelled according to whether the patient acquired the subtype (*) or was colonised on admission (^).

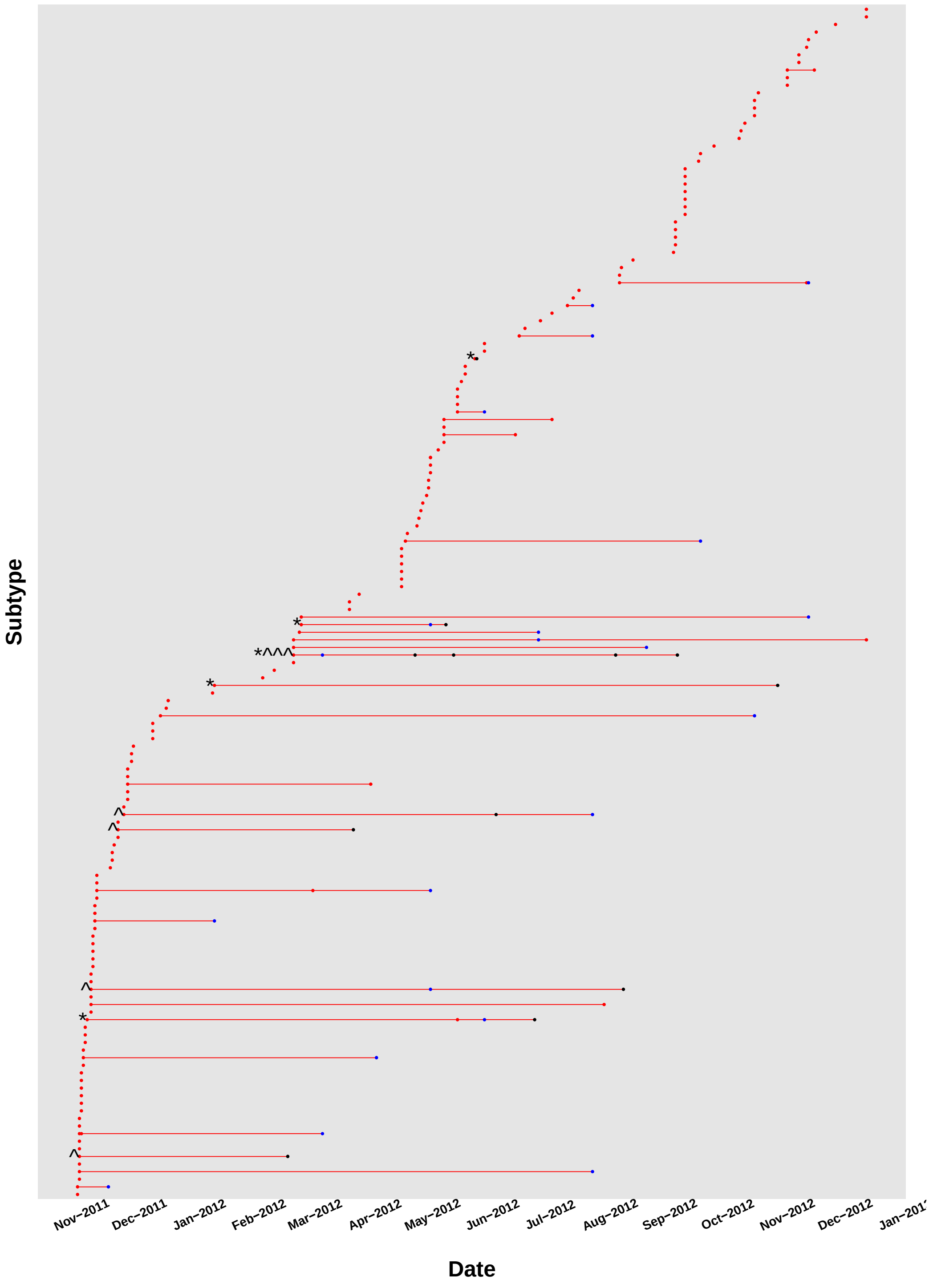

Figure S5. ***Staphylococcus aureus*** **subtypes first identified from patients**

Each row represents a subtype first identified from a patient (n=404). The first isolate from each subtype and source is plotted according to date of collection. Isolates are coloured according to source; health-care worker (HCW) = red, patient = black, environment = blue. Where a subtype was retrieved from >1 of the same source (i.e. >1 patient or HCW) multiple dots of the same colour are plotted. The horizontal lines joining dots of the same subtype are coloured according to the source where each subtype was first identified; HCW = red, environment = blue, patient = black. Subtypes identified in HCWs (n=8) are labelled according to whether the HCW's isolate was identified in the same (or preceding) four-weekly cycle to the patient (*) or whether the HCW cultured the subtype in a four-weekly cycle following the patient (^).

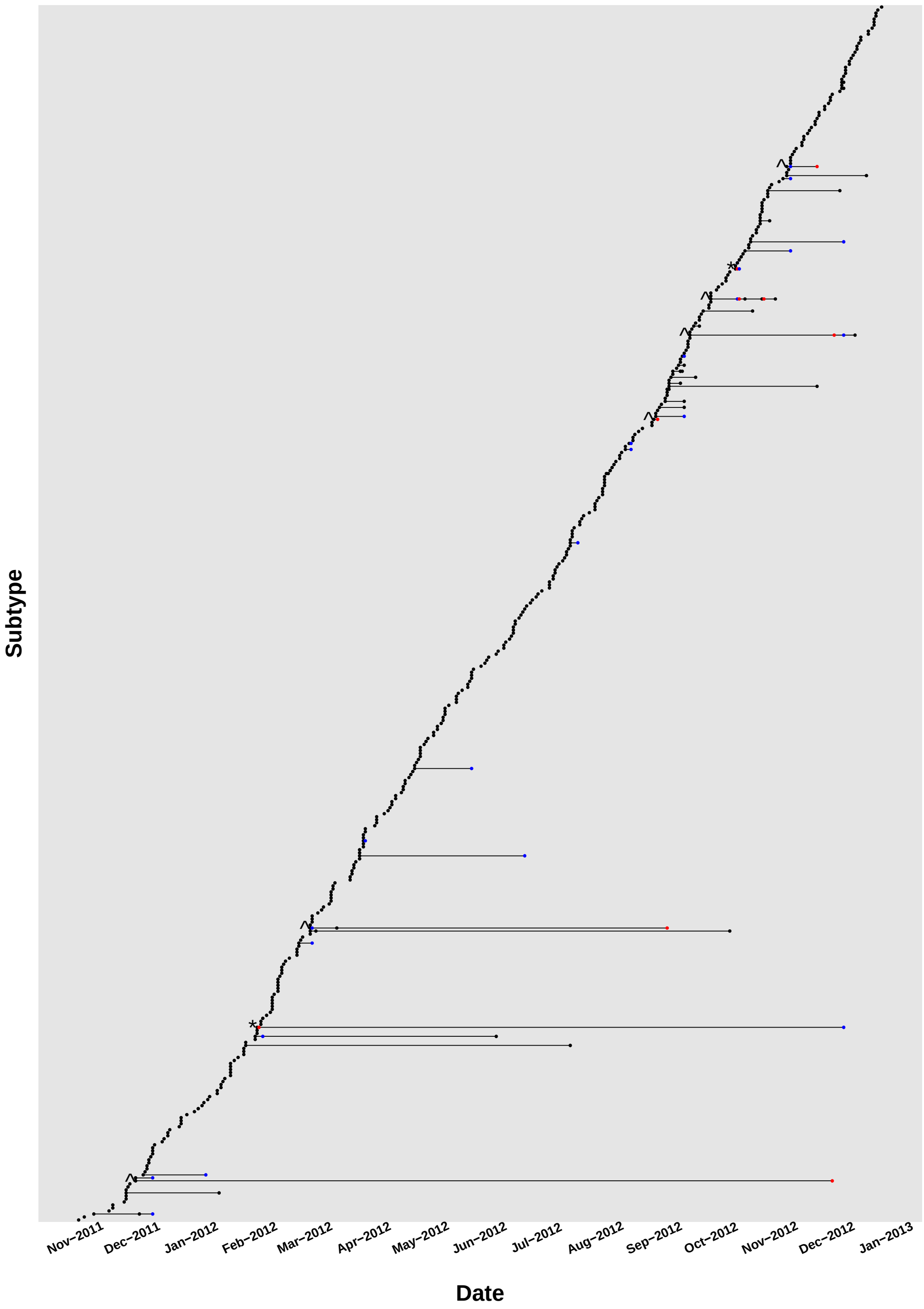

Figure S6. ***Staphylococcus aureus*** **subtypes first identified from an environmental sample**

Each row represents a subtype first identified from an environmental sample (n=44). The first isolate from each subtype and source is plotted according to date of collection. Isolates are coloured according to source; health-care worker (HCW) = red, patient = black, and environment = blue. Where a subtype was retrieved from >1 of the same source (i.e. >1 patient or HCW) multiple dots of the same colour are plotted. The horizontal lines joining dots of the same subtype are coloured according to the source where each subtype was first identified; HCW = red, environment = blue, patient = black.

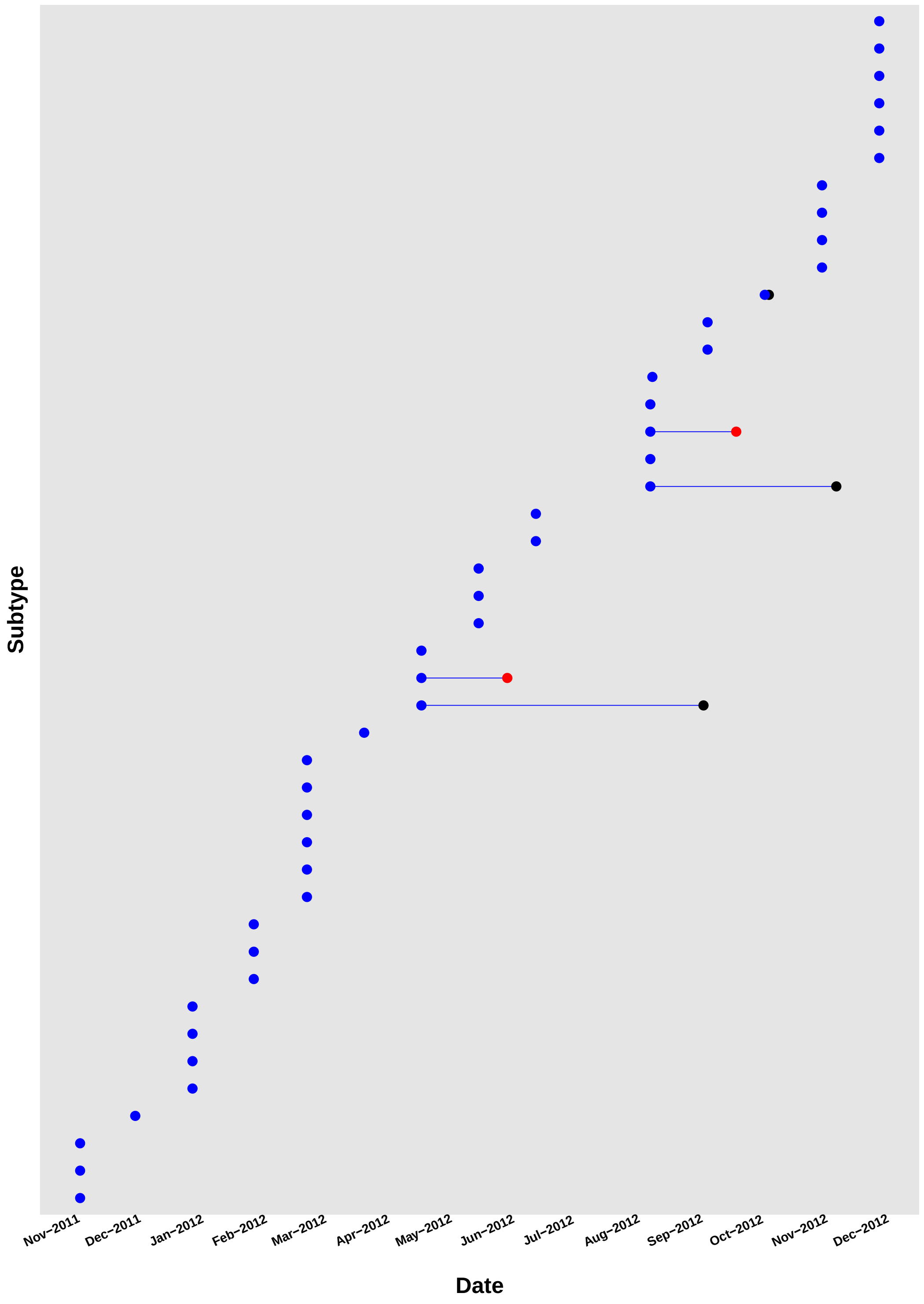

Table S1. Monthly prevalence of *Staphylococcus aureus* in health-care workers and environment

* Denominator is nasal swabs taken from individual health-care workers (HCW) each month; numerator is *S. aureus* positive nasal swabs. †Denominator is air-sampling sites screened each month; numerator is number of *S. aureus* positive air-sampling sites. ‡Denominator is bed-spaces screened each month; numerator is number of *S. aureus* positive bed-spaces. Methicillin resistant *S. aureus* (MRSA).

	**Four-weekly sampling frame**														
	**1**	**2**	**3**	**4**	**5**	**6**	**7**	**8**	**9**	**10**	**11**	**12**	**13**	**14**	**15**
**HCW***															
Total , n (%)	35/96 (36·5)	37/100 (37·0)	37/100 (37·0)	38/103 (36·9)	40/105 (38·1)	33/112 (29·5)	50/139 (36·0)	52/140 (37·1)	50/139 (36·0)	51/140 (35·7)	48/121 (40·0)	52/137 (38·0)	50/134 (37·3)	49/140 (35·0)	42/128 (32·8)
MRSA, n (%)	5/96 (5·2)	6/100 (6·0)	7 /100 (7·0)	8 /103 (7·8)	7/105 (6·7)	5/112 (4·5)	6/139 (4·3)	7/140 (5·0)	7/139 (5·0)	7/140 (5·0)	6/121 (5·0)	5/137 (3·6)	4/134 (3·0)	4/140 (2·9)	6/128 (4·7)
**Air-sampling†**															
Total, n	4/10	2/10	4/10	1/10	5/10	2/10	4/9	5/11	3/11	1/11	4/11	2/11	3/11	5/11	2/11
(%)	(40·0)	(20·0)	(40·0)	(10·0)	(50·0)	(20·0)	(44·4)	(45·5)	(27·3)	(9·1)	(36·4)	(18·2)	(27·3)	(45·5)	(18·2)
MRSA, n	4/10	1/10	0/10	0/10	2/10	1/10	1/9	0/11	0/11	0/11	0/11	0/11	0/11	0/11	0/11
(%)	(40·0)	(10·0)	(0·0)	(0·0)	(20·0)	(10·0)	(11·1)	(0·0)	(0·0)	(0·0)	(0·0)	(0·0)	(0·0)	(0·0)	(0·0)
**Bed-space‡**															
Total, n	4/26	2/26	3/26	3/26	8/26	2/26	6/28	5/28	3/28	6/28	11/28	11/28	7/28	9/27	9/28
(%)	(15·4)	(7·7)	(11·5)	(11·5)	(30·8)	(7·7)	(21·4)	(17·9)	(10·7)	(21·4)	(39·3)	(39·3)	(25·0)	(33·3)	(32·1)
MRSA, n	2/26	0/26	0/26	0/26	0/26	1/26	1/28	0/28	0/28	2/28	0/28	0/28	0/28	0/27	2/28
(%)	(7·7)	(0·0)	(0·0)	(0·0)	(0·0)	(3·8)	(3·6)	(0·0)	(0·0)	(7·1)	(0·0)	(0·0)	(0·0)	(0·0)	(7·1)

Table S2. Routine screens from each patient-admission to the intensive care or high dependency unit

		Screens per admission (n)			Total
		**0**	**1**	**≥2**	
**Admission Duration (days)**	**1**	41	215	59	315
	**2**	17	202	152	371
	**3**	7	131	162	300
	**4**	6	76	128	210
	**5**	2	32	105	139
	**6**	0	26	94	120
	**7**	2	14	92	108
	**8–14**	4	28	207	239
	**15–21**	0	3	68	71
	**22–28**	0	0	26	26
	**29–49**	0	0	28	28
	**≥50**	0	0	6	6
**Total**		79	727	1127	1933

References

1. Andrews J, on behalf of the BSAC Working Party on Susceptibility Testing. BSAC standardized disc susceptibility testing method (version 8). *J Antimicrob Chemother* 2009; **64**: 454-89.

2. Holden MT, Feil EJ, Lindsay JA, et al. Complete genomes of two clinical Staphylococcus aureus strains: Evidence for the rapid evolution of virulence and drug resistance. *Proc Natl Acad Sci U S A* 2004; **101**: 9786-91.

3. Lunter G, Goodson M. Stampy: A statistical algorithm for sensitive and fast mapping of Illumina sequence reads. *Genome Res* 2010; **21**: 936-9.

4. Li H, Handsaker B, Wysoker A, et al. The Sequence Alignment/Map format and SAMtools. *Bioinformatics* 2009; **25**: 2078-9.

5. Didelot X, Wilson DJ. ClonalFrameML: efficient inference of recombination in whole bacterial genomes. *PLoS Comput Biol* 2015; **11**(2): e1004041.

6. Drancourt M, Raoult D. rpoB gene sequence-based identification of Staphylococcus species. *J Clin Microbiol* 2002; **40**(4): 1333-8.

7. Ghebremedhin B, Layer F, Konig W, Konig B. Genetic classification and distinguishing of Staphylococcus species based on different partial gap, 16S rRNA, hsp60, rpoB, sodA, and tuf gene sequences. *J Clin Microbiol* 2008; **46**(3): 1019-25.

8. Holt DC, Holden MT, Tong SY, et al. A very early-branching Staphylococcus aureus lineage lacking the carotenoid pigment staphyloxanthin. *Genome Biol Evol* 2011; **3**: 881-95.

9. McDonald M, Dougall A, Holt D, et al. Use of a single-nucleotide polymorphism genotyping system to demonstrate the unique epidemiology of methicillin-resistant Staphylococcus aureus in remote aboriginal communities. *J Clin Microbiol* 2006; **44**(10): 3720-7.

10. Ruimy R, Angebault C, Djossou F, et al. Are host genetics the predominant determinant of persistent nasal Staphylococcus aureus carriage in humans? *J Infect Dis* 2010; **202**(6): 924-34.

11. Ruimy R, Armand-Lefevre L, Barbier F, et al. Comparisons between geographically diverse samples of carried Staphylococcus aureus. *J Bacteriol* 2009; **191**(18): 5577-83.

12. Ng JW, Holt DC, Lilliebridge RA, et al. Phylogenetically distinct Staphylococcus aureus lineage prevalent among indigenous communities in northern Australia. *J Clin Microbiol* 2009; **47**(7): 2295-300.
